# Sonification as a possible stroke rehabilitation strategy

**DOI:** 10.3389/fnins.2014.00332

**Published:** 2014-10-20

**Authors:** Daniel S. Scholz, Liming Wu, Jonas Pirzer, Johann Schneider, Jens D. Rollnik, Michael Großbach, Eckart O. Altenmüller

**Affiliations:** ^1^Institute of Music Physiology and Musicians' Medicine, University of Music, Drama and MediaHannover, Germany; ^2^Institute for Neurorehabilitational Research (InFo), BDH-Clinic Hessisch Oldendorf, Teaching Hospital of Hannover Medical School (MHH)Hessisch Oldendorf, Germany

**Keywords:** sonification, stroke rehabilitation, auditory-motor integration, pitch perception, timbre perception, music perception, validation of rehabilitation method

## Abstract

Despite cerebral stroke being one of the main causes of acquired impairments of motor skills worldwide, well-established therapies to improve motor functions are sparse. Recently, attempts have been made to improve gross motor rehabilitation by mapping patient movements to sound, termed sonification. Sonification provides additional sensory input, supplementing impaired proprioception. However, to date no established sonification-supported rehabilitation protocol strategy exists. In order to examine and validate the effectiveness of sonification in stroke rehabilitation, we developed a computer program, termed “SonicPointer”: Participants' computer mouse movements were sonified in real-time with complex tones. Tone characteristics were derived from an invisible parameter mapping, overlaid on the computer screen. The parameters were: tone pitch and tone brightness. One parameter varied along the *x*, the other along the *y* axis. The order of parameter assignment to axes was balanced in two blocks between subjects so that each participant performed under both conditions. Subjects were naive to the overlaid parameter mappings and its change between blocks. In each trial a target tone was presented and subjects were instructed to indicate its origin with respect to the overlaid parameter mappings on the screen as quickly and accurately as possible with a mouse click. Twenty-six elderly healthy participants were tested. Required time and two-dimensional accuracy were recorded. Trial duration times and learning curves were derived. We hypothesized that subjects performed in one of the two parameter-to-axis–mappings better, indicating the most natural sonification. Generally, subjects' localizing performance was better on the pitch axis as compared to the brightness axis. Furthermore, the learning curves were steepest when pitch was mapped onto the vertical and brightness onto the horizontal axis. This seems to be the optimal constellation for this two-dimensional sonification.

## Introduction

Impairments of motor control of the upper limbs are frequently the consequences of a stroke. Numerous training approaches have been designed, addressing different aspects of sensory-motor rehabilitation. For example, intensive practice of the disabled arm leads to a clear improvement, which is even more pronounced when the unimpaired limb is immobilized. However, this constraint-induced movement therapy (Taub et al., 1999),—albeit efficient (Hakkennes and Keating, 2005; Peurala et al., 2012; Stevenson et al., 2012)—is not always very motivating and may even lead to increased stress and thus sometimes fails to improve the mood and the overall quality of life of patients due to the nature of the intervention (Pulman et al., 2013). Alternatively, training programs using playful interactions in video games (Joo et al., 2010; Hijmans et al., 2011; Neil et al., 2013) point at the possibility to utilize multisensory visual-motor-convergence in order to improve motor control. Again, these rehabilitation strategies, although more motivating, have not yet gained wide acceptance in rehabilitation units (Lohse et al., 2014). Here, the possibility of using auditory information supplementary to visual feedback in order to inform patients about movements of their impaired arms is a promising new method, referred to as “sonification.” More generally speaking, sonification denotes the usage of non-speech audio to represent information, which is otherwise not audible (Kramer et al., 1999).

In a pilot project conducted together with colleagues from the departments of Sports Education and Microelectronic Systems of the Leibniz University Hannover, a portable sonification device suitable for real-time music-based sonification in a stroke rehabilitation setting was developed. This 3D sonification device is going to be evaluated in a larger stroke patient population following the preliminary 2D experiment presented in this paper. The long-term goal of this research project is to improve the rehabilitation of gross-motor arm skills in stroke patients by attaching small sensors to the arm and thereby sonifying movements onto a 3-dimensional sound map, using basic musical parameters to inform patients acoustically about the position of their impaired arm in space. The rationale behind this approach was the idea of taking advantage of three important mechanisms driving neuroplasticity. Generalizing, these three mechanisms could also be subsumed under “enriched environment conditions” for stroke patients (Eng et al., 2014). First, we believe that the emotional and motivational power of music may reinforce learning processes by making the patients compose and play “tunes” in a playful manner when moving their impaired limb (Koelsch, 2005; Croom, 2012; Karageorghis and Priest, 2012a,b; Bood et al., 2013), second, sonification may replace deteriorated proprioceptive feedback (Sacco et al., 1987) and third, sonification supports auditory sensory-motor integration by establishing brain networks facilitating the transformation of sound into movement (Paus et al., 1996; Bangert and Altenmüller, 2003; Bangert et al., 2006; Altenmüller et al., 2009; Scheef et al., 2009; Andoh and Zatorre, 2012). In order to find the most effective and intuitive musical sonification therapy, it is important to clarify how movements in different spatial dimensions should best be musically mapped in space. Dubus and Bresin (2013) reviewed 60 sonification research projects and found in most of them verticality to be associated with pitch. However, for example in pianists, pitch is associated with horizontality. Walker (2007) developed an important framework for sonification. He found that three design decisions are critical when applying sonification. First, it is crucial which sound dimension should represent a given data dimension. Second, an appropriate polarity for the data-to-display mappings has to be chosen. Third, the scaling of the mapping has to be carefully adjusted to the respective needs. For example fine motor movements of the fingers require a different scale of sound mapping as compared for example to sonification of gait.

In the present study we limited ourselves to two sound parameters namely pitch and brightness and therefore to two dimensions. Furthermore we tested only one polarity each, since we were interested in whether pitch or brightness should be on the vertical movement axis. For this aim, we mapped pitch either vertically rising from bottom (“low”) to top (“high”), since this is how it is used in our daily language. Or horizontally from left (“low”) to right (“high”) comparable to a conventional piano. Brightness was mapped horizontally from left (“dull”) to right (“bright”), comparably to turning an equalizer knob clockwise, so that the sound becomes brighter. We used the sonification dimensions pitch and brightness since in many acoustical musical instruments they can be directly manipulated by the player. Furthermore we used discrete tonal steps from the diatonic system because we wanted the participants to play simple tunes later on without practicing the more demanding intonation first.

We intended to investigate the subjects' ability to infer spatial origin of a sound from acoustic information varying in the mentioned parameters by establishing an implicit knowledge of the current parameter-to-axis mappings.

## Materials and methods

### Participants

Twenty-six healthy subjects (13 women) participated in this study. All subjects, aged between 52 and 82 years (*M* = 65.8; *SD* = 8.6) were recruited from homes for the elderly and at social events for older people in Hanover, Germany. We explicitly looked for subjects aged between 50 and 80 years to address a population whose age is comparable to the population with highest risk of stroke. All subjects were right-handed and had no neurological or psychiatric disorders. They all had normal hearing abilities, according to testing with test tones before starting the experiment. If necessary, sound volume was adjusted. They had no mobility limitations in the right shoulder or arm. All of the subjects had some prior musical education (e.g., music lessons at school, choir singing for 3 years, or 5 years of piano-lessons some 20 years ago, respectively), however, none of the subjects were professional musicians, or had had experience with the experiment or a similar task before. The subjects were randomized to start with one of two different experimental conditions, 1 or 2.

### Experimental setup

Stimulus presentation and user interaction was accomplished with a custom-made program written in Puredata (PD, http://puredata.info), an open source programming environment running on a computer under a Debian Linux operating system (version 7, “wheezy”). A standard LCD screen and a USB mouse were used. Sound was conveyed via headphones (Sennheiser HD 437).

### Procedure

All sounds were synthesized in Pure Data as complex tones consisting of fundamental sine-tones plus added harmonics.

**Figure 1:**
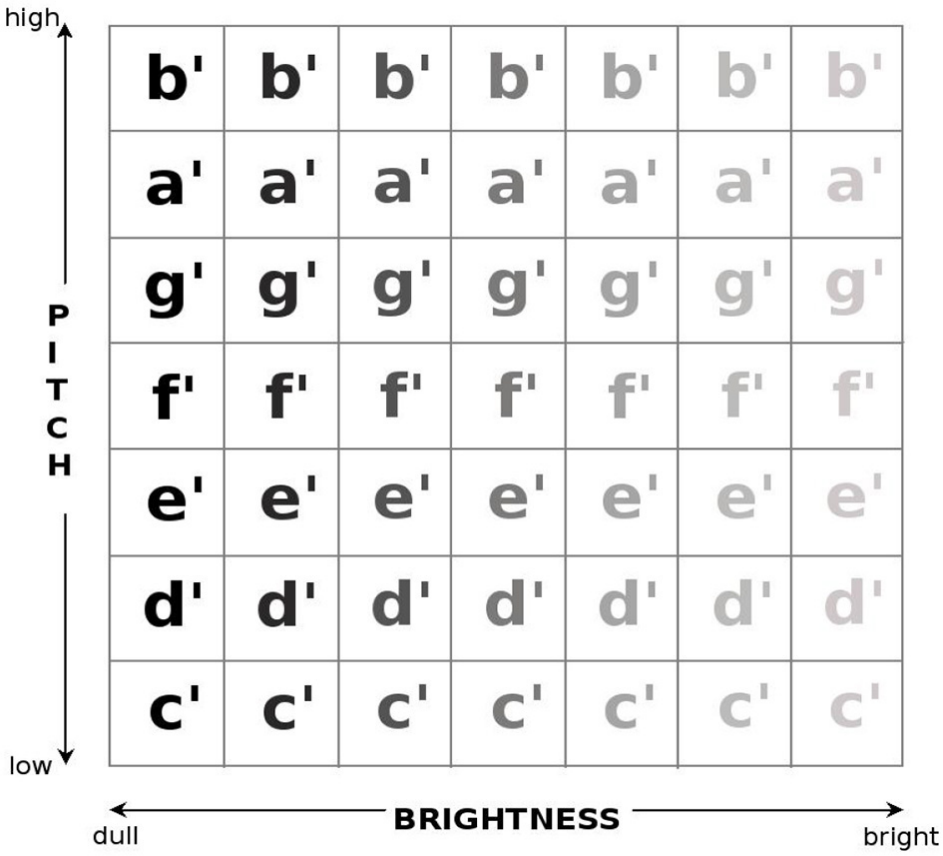
**Invisible overlaid 7 × 7 matrix of the sound parameters mapped onto a plane**. Condition 1, in which pitch was mapped onto the *y* axis and brightness onto the *x* axis is shown. In condition 2 the square parameter grid was rotated clockwise by 90° putting pitch onto the *x* axis and brightness onto the *y* axis.

The stimulus for a given trial was synthesized online, using one pseudo-randomly picked square from an invisible 7 × 7 grid overlaid on the screen as the spatial origin of the sound. The current mapping of the sound parameters to the spatial *x* and *y*-axes was used to determine the pitch (fundamental frequency) and brightness (number of overtones) of the sound (Figure 1). Since parts of the experimental setup will later be used in a stroke rehabilitation setting for gross motor skills, resolution was limited to the small number of seven discrete steps, thus being comparable to a diatonic acoustical musical instrument on which one can play simple songs.

Subjects were seated comfortably on a height-adjustable chair in front of a desk, and the experimenter read out standardized instructions. Subsequently, the program was started and the subjects were presented with the exploration trial and the instruction “*please explore the screen with the mouse*.” Moving the mouse cursor resulted in the sound changing according to the overlaid grid and the current condition (Figure 1). This feedback served for the subjects to build-up implicit rules of the relationship between spatial coordinates of the mouse cursor and the resulting sound parameters.

**Figure 2:**
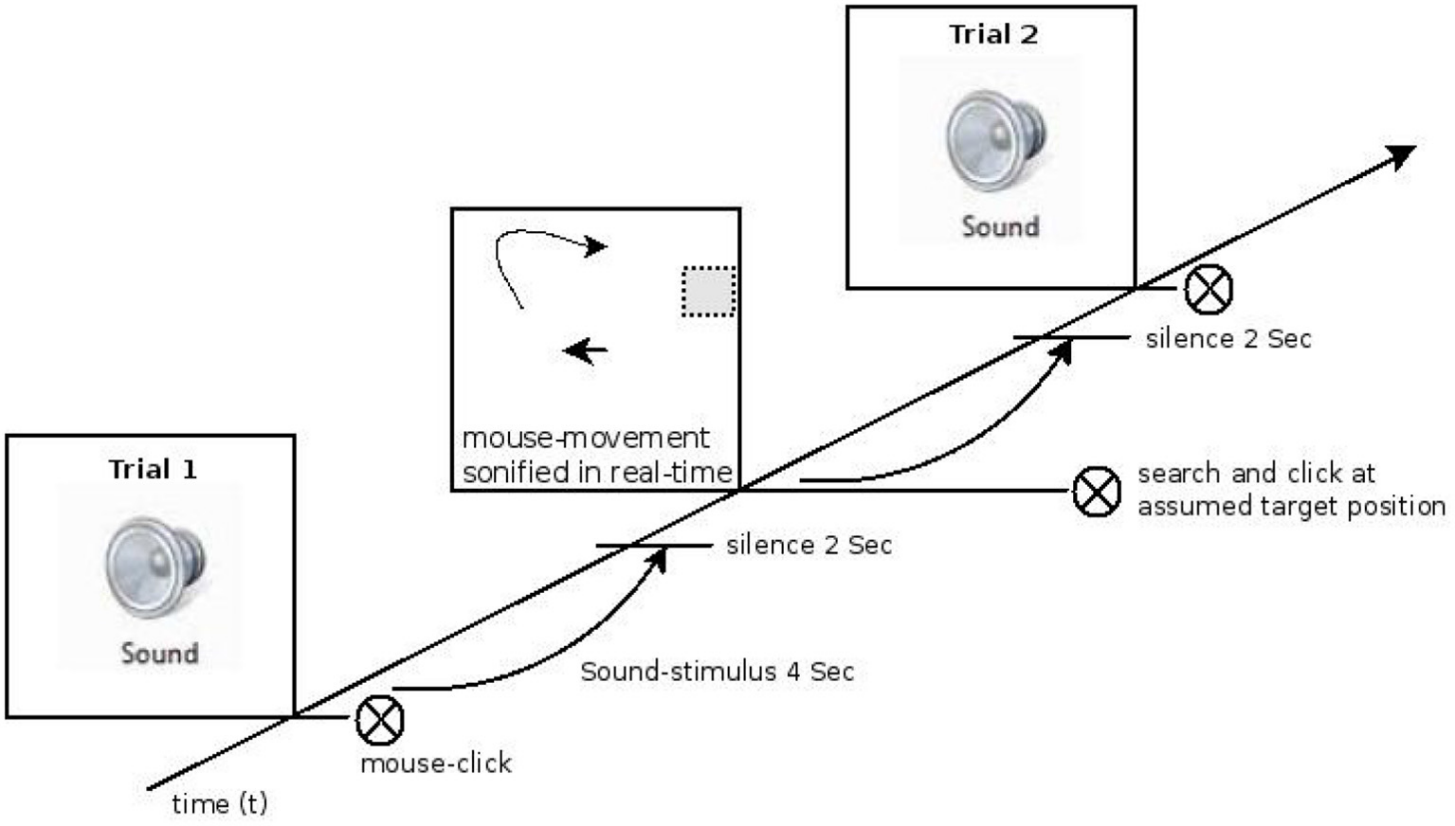
**Schematic timeline of one experimental block**. In the left lower quadrant, the exposure phase when the sound stimulus was presented is shown. This was followed by the “finding-phase” in which the subject searched the origin of the previously heard but on the screen invisible target sound stimulus using the sonified mouse movement as feedback. Finally the subjects clicked at the supposed target position.

During the actual test subjects saw a white screen and were presented with a sound for 4 s. The presentation of the stimulus was followed by a pause of 2 s. Subjects then were instructed to move the mouse cursor to the position on the screen where they felt the sound might have originated from based on their experience from the exploration phase. During the subsequent mouse movement the sound output changed in real-time according to the current mapping rules of pitch and brightness and the position of the mouse cursor. Subjects could use this feedback to compare their working memory trace of the target sound with the current position's sound. Subjects were asked to click the mouse at the position they felt the initial stimulus had been derived from as fast but as precisely as possible. Figure 2 shows the experimental procedure. The entire test consisted of 100 trials (lasting about 40 min in total), subdivided into 50 trials of *condition. 1*, a 10s-break in-between, and 50 trials of *condition 2*. Pitch and brightness mappings onto the two axes were presented in two blocks with the order balanced across subjects.

#### Condition 1

Pitch was mapped as a C-major scale onto the *y* axis ranging from c′ (261.6 Hz in Helmholtz pitch notation) at the bottom to b′ (493.9 Hz) at the top of the screen. Brightness was mapped onto the *x* axis (also in seven steps), and realized by a bandpass filter allowing harmonics ranging from 250 to 1250 Hz to either pass or not pass, resulting in a very bright sound on the right side of the screen and a dull sound at the left. This brightness filter works comparably to an equalizer so that e.g., on the very left the sound is very dull and on the very right the sound is very bright (Figure 1).

#### Condition 2

In condition 2 the parameter grid was rotated clockwise by 90°. Brightness was then mapped onto the *y* axis and pitch onto the *x* axis. The subjects could never see, only hear the borders between the 49 fields of the grid, and they were naive to the fact that parameter mappings changed between experimental blocks and were never told which sound parameters were manipulated.

### Measurements

The dependent variables were “Time” and “Click-Target Distance.” “Time” denotes the duration it took the subject to search and indicate the spatial origin of the sound stimulus by a mouse click. Click-Target Distance was derived as the city block distance between the mouse click and the target field on the grid. A trial was considered a full “hit” when the subject had clicked on the grid cell in which both brightness and pitch matched perfectly the previously heard sound stimulus.

### Data analysis

Raw data were collected with Puredata and then processed with Python 2.7 scripts (www.python.org). Statistical analysis was conducted using R (http://www.r-project.org, version 3.0.1) in RStudio (http://www.rstudio.com, version 0.97.551). Trial duration times were recorded. Shapiro Wilks tests showed that data were not normally distributed. Outlier elimination was conducted by removing trial duration times further away than 2.5 standard-deviations from the median. Trial duration times were binned into five 10-trial blocks for each condition and plotted in boxplots. Friedman tests were conducted to check whether there was a significant change in trial duration times over time. One-sided paired Wilcoxon tests were used as post-hoc tests to detect a potential decrease of trial duration time over time. Bonferroni correction was used to prevent alpha inflation. A F-test was calculated to compare the variances of the trial duration times for both conditions. City block distances between click and target fields were calculated separately for *x* and *y* mouse coordinates. Shapiro–Wilks tests showed that data were not normally distributed. Trials were binned into five 10-trial blocks to show a potential learning effect over time. Means, standard-errors and confidence intervals for the bins were calculated. Friedman tests were conducted to check whether there was a significant change of mean click-to-target distance over time. One-sided paired Wilcoxon-tests were used as post-hoc tests to detect whether there was a significantly smaller mean click-to-target distance in the last trial bin (#5) as compared to the first trial bin mean (#1) of a given mapping. Again Bonferroni correction was used to prevent alpha inflation. Paired Wilcoxon signed-rank tests were performed to compare the learning curves and the effectiveness of the mappings within and across the two conditions. Due to incomplete data, two participants had to be excluded from further analysis.

## Results

### Trial duration time

#### Condition 1

**Figure 3:**
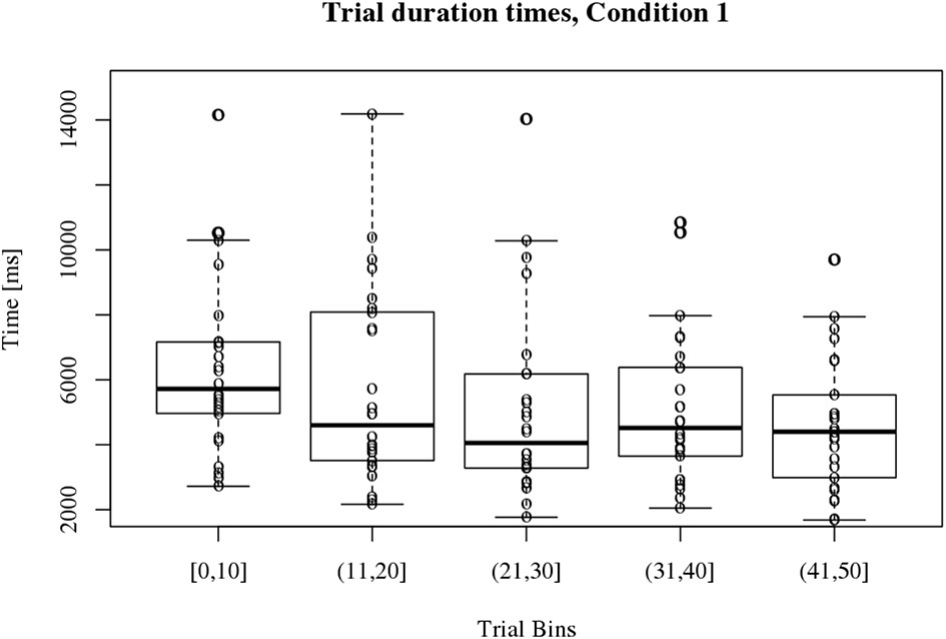
**Boxplots of participant's trial duration times for condition 1**. The 50 trials were binned into 5 bins of 10 trials as shown on the *x* axis. Participant's trial duration times vary around 5000 ms as depicted on the *y* axis. There is a significant decrease of participant's trial duration times over time (^**^p < 0.001) when comparing Bin #1 and Bin #5.

Figure 3 displays participants' trial duration times over time for condition 1. Participant's times varied around 5000 ms. The medians for the trial duration time bins of condition 1 are significantly different [χ^2^_(4)_ = 29.8, *p* < 0.001]. The paired Wilcoxon *post-hoc* test revealed a significant reduction of trial duration time over time for condition 1 when comparing bin #1 and #5 (*V* = 343, *p* < 0.001).

#### Condition 2

**Figure 4:**
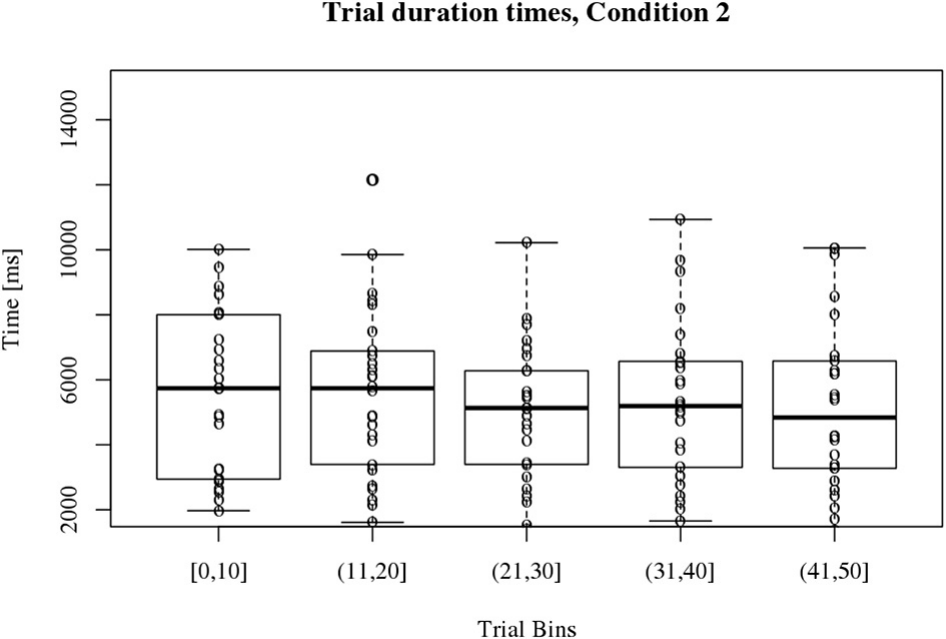
**Boxplots of participant's trial duration times for condition 2**. The 50 trials were binned into 5 bins of 10 trials as shown on the *x* axis. Trial duration times vary around 5000 ms as depicted on the *y* axis. There is no significant change over time.

Figure 4 displays participants' trial duration times for condition 2 which also varied around 5000 ms. Participant's trial duration times for condition 2 are more homogeneous and the medians for the bins are not significantly different over time [χ^2^_(4)_ = 5.04, *p* = 0.28]. Therefore, no *post-hoc* evaluation seemed necessary.

#### Comparison of the trial duration times for condition 1 and 2

Overall participant's trial duration times for condition 1 are more heterogeneous than for condition 2 which can be derived from the boxplots in Figures 3, 4. Participants become significantly faster in clicking at the assumed target position in condition 1. This is not the case for condition 2.

No significant difference of the variances of the trial duration times for condition 1 and 2 was found [*F*_(129, 129)_ = 1.21, *p* = 0.28].

### Learning curves

#### Condition 1

**Figure 5:**
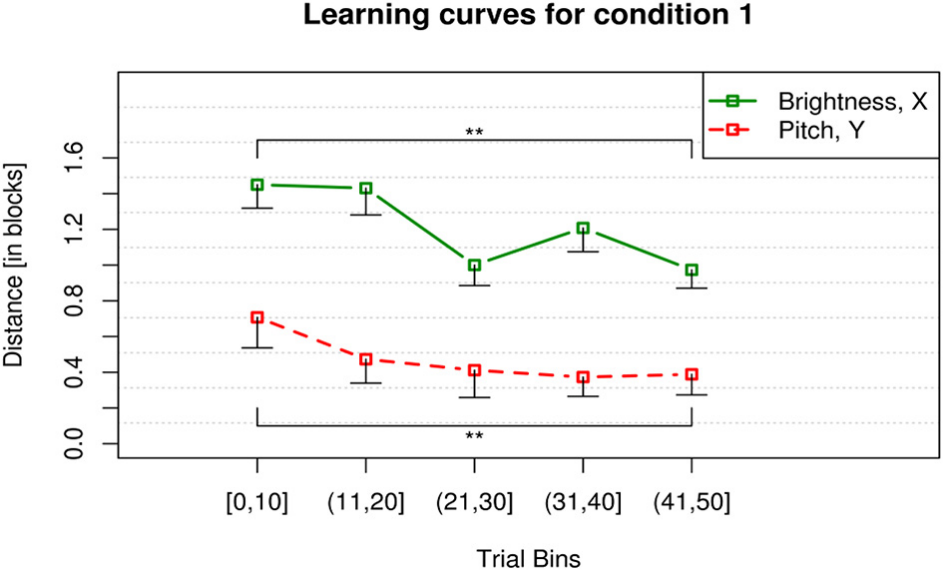
**Learning curves for condition 1**. The *y* axis displays the mean city-block distance between participants' clicks and the target for *x* and *y* position of the mouse. For the *x* axis, the 50 trials of each participant within a block were binned into 5 bins of 10 trials and averaged across participants. The error bars display the lower 99 % confidence boundary below participants' mean click-to-target distance in the respective trial bin. Participants showed a significant decrease of click-to-target distance for both dimensions, i.e., pitch (red, dashed) and brightness (green, solid) in the course of the condition. ^**^p < 0.001.

In condition 1, when pitch was mapped onto the *y* axis and brightness was mapped onto the *x* axis, participants showed a significant learning effect for the parameter pitch [χ^2^_(4)_ = 34.06, *p* < 0.001]. Learning can be assumed if the distance from participants' clicks to the target coordinates decreases over time (Figure 5). The mean click-to-target distance was lower at the end (Bin #5) as compared to the beginning (Bin #1) as shown by the results of the Wilcoxon *post-hoc* test (*V* = 285.5, *p* < 0.001). A significant decrease of click-to-target distance for the parameter brightness [χ^2^_(4)_ = 13.14, *p* < 0.01] (*V* = 175, *p* = 0.005) was also shown in condition 1. The overall click-to-target distance for brightness was higher than for pitch which can be seen in Figure 5. The paired Wilcoxon signed-rank test showed that pitch was the more effective mapping in condition 1 (*V* = 540.5, *p* < 0.001).

#### Condition 2

**Figure 6:**
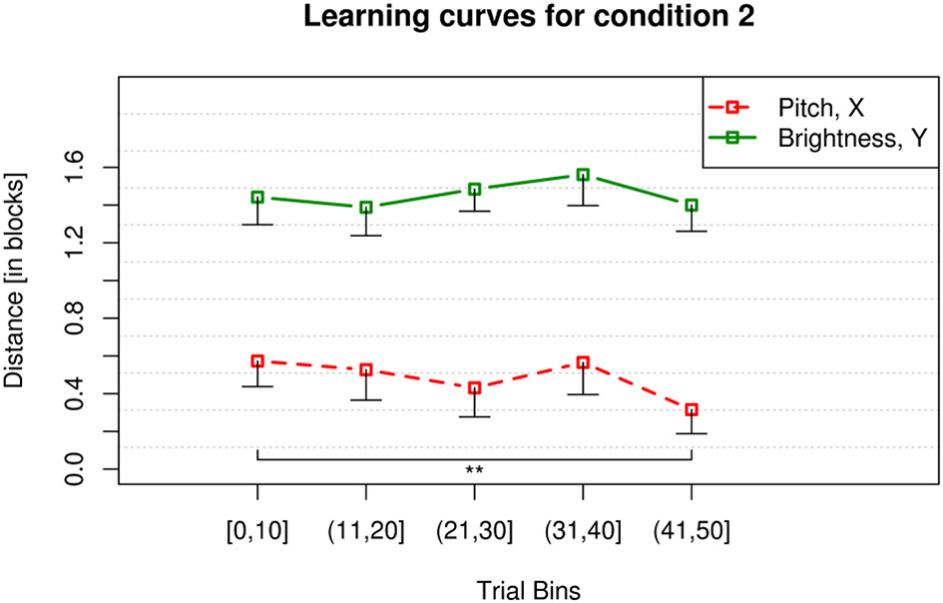
**Learning curves for condition 2**. The *y* axis displays the mean city-block distance between participants' clicks and the target for *x* and *y* position of the mouse. The 50 trials were binned into 5 bins of 10 trials as shown on the *x* axis. The error bars display the lower boundary of a 99 % confidence interval below participants mean click-to-target distance in the corresponding trial bin. Participants showed a significant decrease of click-to-target distance over time for the dimension pitch (red, dashed) but not for brightness (green, solid). ^**^p < 0.01.

For condition 2 the sound parameter grid was rotated, mapping brightness onto the *y* axis and pitch onto the *x* axis. Participants showed a significant learning effect for the parameter pitch displayed by a significant reduction of click-to-target distance over time [χ^2^_(4)_ = 21.52, *p* < 0.001] (*V* = 182.5, *p* = 0.002) (Figure 6). They did not show a significant reduction of click-to-target distance over time for the parameter brightness [χ^2^_(4)_ = 7.15, *p* = 0.128]. Also in condition 2 the click-to-target distances of the participants for brightness were always higher than for pitch. Participants were always further away from the goal for brightness than for pitch. So in condition 2 pitch was again the more effective mapping as displayed in Figure 6 and by the results of the paired Wilcoxon signed-rank test (*V* = 7896.5, *p* < 0.001).

#### Comparison of the learning curves for condition 1 and 2

The performance measured in blocks for the dimension pitch across conditions 1 and 2 is not significantly different (*V* = 1875, *p* = 0.603) (see red dashed lines in Figures 5, 6, respectively) when tested binwise. This means participants learn pitch in both conditions equally well and with a comparable progress. In both conditions their click-to-target distance for the parameter pitch is significantly reduced toward the end of the 50 trials as compared to the beginning. Pitch was in both conditions the more effective mapping displayed by overall less click-to-target distance of the participants.

Whereas the performance for the dimension brightness over the two conditions is significantly different (*V* = 5490.5, *p* < 0.001) (see green solid lines in Figures 5, 6, respectively). Indicating that brightness is learned well when being mapped onto the horizontal axis. Brightness is not learned at all when being mapped vertically.

## Discussion

The aim of this study was to optimize a movement-to-sound mapping using musical stimuli, since there is a lack of objective evaluation of sonification parameters (Dubus and Bresin, 2011). In succession of this study Dubus and Bresin (2013) reviewed 60 sonification research projects and found that only in a marginal number of them the sonification mappings had been carefully assessed in advance. This review therefore stresses the need for validation of sonification parameter mappings as conducted in the present study.

The results of the present study are that (1) participants become faster in finding the goal when pitch is being mapped onto the vertical movement axis and brightness is being mapped onto the horizontal movement axis. (2) They learn both dimensions well if the mappings are the aforementioned. (3) Pitch is generally learned well and more precisely. Pitch is the more effective mapping in both conditions. Brightness is only learned well when being mapped onto the horizontal movement axis.

These results imply that (1) the choice of the axes is critical and (2) pitch is better matched on the vertical axis. This is also in line with the review of Dubus and Bresin (2013), who found verticality to be associated in most of the sonification paradigms with pitch.

When addressing the pioneering framework to evaluate sonification mappings of Walker (2007), our long-term goal was to enable stroke patients to produce simple folksong melodies with their arm movements. It was therefore mandatory to introduce a sonification mapping with discrete steps. In the present study, this approach was chosen in order to avoid the more difficult practice of intonation first and to enable patients to play easy folk-songs in tune and with the correct intervals from the beginning on. We strove to render the sonification as intuitive and as much as possible comparable to an acoustical musical instrument. This is one of the major differences between our and other sonification design approaches in this field (e.g., Chen et al., 2006). Later, we will encourage the patients to actively play and create music by their movements. By doing that sound will not only be a passive byproduct of e.g., a grasping motion. Our sonification training will be designed to resemble a music lesson rather than a shaping of movements while sound is being played back.

In the present experiment we focused for simplicity reasons on two (pitch, brightness) out of three sonification dimensions (pitch, brightness, volume) which will be used in a later study. A 3D mapping was too complicated for our elderly subjects, not used to work with interactive computer programs. We decided to map brightness and pitch only in one polarity because the main question of this study was to find out whether either pitch or brightness should be mapped on the vertical movement axis. Additionally, the trial number would have had to be doubled when permuting two polarities of two dimensions to gain sufficient statistical power. The experiment already took 45 min for only two dimensions with one polarity each and subject's concentration was highly committed to the demanding task.

We used a novel approach by introducing musical stimuli such as a musical major scale with discrete intervals and timbre parameters derived from the sound characteristics of acoustical musical instruments.

One of the ideas was that participants could improve control of arm positions in space via associative learning, leading to associating a given relative arm position with a specific musical sound. This sound-location association might then substitute the frequently declined or even lost proprioception. Additionally, the trajectories while moving their arms to the target point would be audible as well. Thus multimodal learning could take place because subjects are being provided with sound as an extra dimension supplying information.

In view of further clinical application, reduced gross motor functions of the arm and reduced proprioception (Sacco et al., 1987) are common disabilities in stroke patients. Hence, the advantages of continuous real-time musical feedback are first aiming at the retraining of gross motor movements of the arm, which are the most disabling challenges in early rehabilitation of stroke. Second, real-time sonification may substitute deficits in proprioception of the arm, which frequently are a consequence of stroke.

Finally we will use the advantage of a highly motivating way to transform movements into sound and thus enhance emotional well-being through the creative, playful character of such a rehabilitation device (Koelsch, 2005, 2009; Eschrich et al., 2008; Croom, 2012; Bood et al., 2013).

In contrast to brightness, pitch is more salient and has a strong spatial connotation in everyday life. This is reflected in language, denoting sounds as “high” or “low” as an example for an implicit visuo-auditory synesthetic concept. However, we have to keep in mind that the same holds for timbre, since “brighter” or “darker” sounds are adjectives taken from the visual domain. One could even argue that a mapping of brightness on the vertical axis could be understood as a metaphor for the brightness shift of an evening or morning sky. Vice versa, pitch mapping onto the *x* axis could be conceptualized as the mapping of conventional piano scales, placing high notes on the right and low notes on the left part of a keyboard, a distribution familiar also to non-musicians. Therefore, showing that the way how sonification parameters are mapped in space is crucial and not a trivial finding. Furthermore, we could exclude order and exhaustion effects by randomizing the mapping order.

Taken together, we have defined a useful spatial mapping of musical sound parameters, applicable in elderly non-musicians and supporting learning effects in auditory sensory-motor integration. This will be the starting point to implement multimodal learning of spatial, motor, auditory, and proprioceptive information in rehabilitation of arm motor control in stroke patients.

### Conflict of interest statement

The authors declare that the research was conducted in the absence of any commercial or financial relationships that could be construed as a potential conflict of interest.
